# Editorial: Bubbles, Droplets and Micelles for Acoustically-Mediated Drug/Gene Delivery

**DOI:** 10.3389/fphar.2020.00954

**Published:** 2020-06-26

**Authors:** Jean-Michel Escoffre, Baudouin Denis de Senneville, Noboru Sasaki, Marc Derieppe

**Affiliations:** ^1^ UMR 1253, iBrain, Université de Tours, Inserm, Tours, France; ^2^ Institut de Mathématiques de Bordeaux (IMB), UMR 5251 CNRS/Université de Bordeaux, Talence, France; ^3^ Laboratory of Veterinary Internal Medicine, Department of Clinical Sciences, Graduate School of Veterinary Medicine, Hokkaido University, Sapporo, Japan; ^4^ Department of Pediatric Neuro-oncology, Princess Maxima Center for Pediatric Oncology, Utrecht, Netherlands

**Keywords:** ultrasound, bubble, therapeutic ultrasound, drug delivery, gene delivery

This special issue presents novel contributions from 86 authors in a compilation of nine original articles and two review articles: four articles from Asia, three from America, and four from Europe. The issue presents findings in the fields of bactericidal therapy, cancer, inner-ear diseases, and underlying mechanisms of ultrasound-mediated drug/gene delivery, all of them with a clear goal: clinical translation. All findings reported in this issue had 8,517 views on April 10^th^ 2020 (source of counting: Frontiers in Pharmacology).

Therapeutic ultrasound shows promising findings in ultrasound-mediated nitric oxide (NO) delivery using lipid-shelled nitric oxide-loaded microbubbles. NO is a potent bioactive gas that was evidenced to display biofilm dispersion and bactericide properties; its delivery in specific anatomical regions is increasingly investigated (Elnaggar et al., 2017), and paves the way for novel encapsulation formulations. In this issue, Lafond and colleagues report on an *in vitro* proof-of-concept demonstrating the relevance to co-encapsulate octafluoropropane in a microbubble formulation containing NO (Lafond et al.). The authors showed an increased payload loading, compatible acoustic properties using a clinical ultrasound scanner, and a significant increase in bacterial killing.

For cancer applications the interest in acoustic cluster therapy (ACT) for enhanced drug/drug carrier delivery is rising (Sontum et al., 2015). Here, two articles evidence enhanced therapeutic effect *in vivo*, 1) for the treatment of triple negative breast cancer using the stealth liposomal doxorubicin, Doxil^®^ (Bush et al.), and 2) for human colon cancer treatment with combined irinotecan (Bush et al.).

An interesting article reports on promising data for future treatments of inner ear diseases (Lin et al.). The authors evidenced the possibility to perform drug delivery to the inner ear non-invasively by ultrasound- and microbubble-mediated permeabilization of the round window membrane. Preservation of the inner ear, this vulnerable and poorly-accessible sensory organ, was clearly documented by not only functional assessment, using auditory brainstem response recordings, but also a morphological evaluation with electron microscopy. To our knowledge, Lin and colleagues are the first to show ultrastructural changes of the round window membrane after its ultrasound- and microbubble-mediated permeabilization. Specifically, the ultrasound protocol applied in this study evidenced that the observed damages did not affect the basement membrane, thus allowing epithelial regeneration. We look forward to consulting future results from this research line.

After around 15 years of fundamental findings and technical developments in focused ultrasound (FUS) for blood-brain barrier (BBB) opening (Hynynen et al., 2001), a few clinical studies using commercially-available FUS systems were published in the last 5 years (Carpentier et al., 2016) (Lipsman et al., 2018) (Idbaih et al., 2019) (Mainprize et al., 2019), thus confirming its potential to give a wealth of molecules and delivery systems, *e.g.,* nanomedicines and viral vectors, access to the parenchyma of the central nervous system (CNS). In this issue, Fisher and Price stress the relevance to combine FUS-mediated BBB opening and polymeric or lipid-based nanoparticles for drug and gene delivery to 1) make therapeutic advances in CNS disorders, 2) offer new opportunities in the detection of early biomarkers, for instance using FUS-mediated BBB opening for antibody delivery in the CNS, or 3) adopt novel approaches to uncover normal and diseased brain function, like targeted Propofol delivery to the thalamus to elicit and study functional changes in rat brain activity (Wang et al., 2018). The authors explain how specific delivery system formulations, *e.g.*, poly (aspartic acid)—polyethylene glycol (PAA-PEG) nanoparticles, can display favorable pharmacokinetic profiles and lead to increased therapeutic effect (Timbie et al., 2017).

Overall, this special issue brings a collection of research articles that seemingly contribute to the scientific advances in the different aspects of bubbles, droplets, and micelles for acoustically-mediated drug/gene delivery, from the formulation of acoustic-responsive agents to preclinical and clinical investigations. The rise of these therapeutic ultrasound applications is the result of longstanding collaborations between academic and private stakeholders of fundamental and applied research who join forces in a multidisciplinary landscape. To fulfill the medical needs is just a matter of time.

## Author Contributions

All four authors organized the Research Topic, invited authors, participated in the review process of the manuscripts, and wrote the editorial.

## Conflict of Interest

The authors declare that the research was conducted in the absence of any commercial or financial relationships that could be construed as a potential conflict of interest.
